# Determination of Atenolol and Trimetazidine in Pharmaceutical Tablets and Human Urine Using a High Performance Liquid Chromatography-Photo Diode Array Detection Method

**DOI:** 10.1155/2019/9625849

**Published:** 2019-01-03

**Authors:** Walaa El-Alfy, Omnia A. Ismaiel, Magda Y. El-Mammli, Abdalla Shalaby

**Affiliations:** Department of Analytical Chemistry, Faculty of Pharmacy, Zagazig University, Zagazig 44519, Egypt

## Abstract

A simple RP-HPLC-PDA method for determination of atenolol (ATN) and trimetazidine (TMZ) in human urine and tablets has been developed. Analytes were separated on a Caltrex BI column (125× 4.0 mm, 5 *μ*m) with 25mM potassium dihydrogen phosphate pH 3.3, methanol, and acetonitrile mobile phases. The PDA detector was operated at 210 nm for TMZ and 225 nm for ATN and the flow rate was 1.0 mL/ min. Linearity was obtained over a concentration range of (1.0-100 *μ*g/mL) for both analytes in standard solutions and the method was successfully applied for determination of target analytes in their pharmaceutical tablets. Excellent linearity was also obtained over concentration ranges of (0.25-25 *μ*g/mL) and (0.5-25 *μ*g/mL) in human urine for TMZ and ATN, respectively. A simple liquid-liquid extraction was applied for urine sample clean-up and a gradient method was used for chromatographic separation. The lower limit of quantitation (LOQ) was 0.99 and 0.60 *μ*g/mL for ATN and TMZ, respectively. The limit of detection (LOD) was 0.30 and 0.18 *μ*g/mL for ATN and TMZ, respectively. Inter- and intraday precision and accuracy for ATN were within ±1.89% in pure form and within ±2.85% in urine samples. Inter- and intraday precision and accuracy for TMZ were within ± 3.99% in pure form and within ± 3.19% in urine samples.

## 1. Introduction

**Figure 1:**
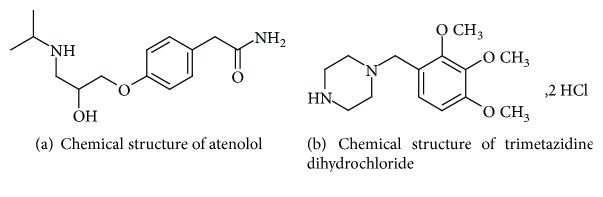


Atenolol (ATN) (4-(2-hydroxy-3-isopropylamino propoxy) phenyl acetamide) (Figure 1) is a cardioselective *β*-blocker compound; it is documented officially in BP [1] and USP [2]. It has been reported that ATN suppresses the release of rennin and angiotensin-II and lowers the aldosterone production [3]. It has neither intrinsic sympathomimetic nor membrane stabilizing actions. It is clinically used in controlling of hypertension, cardiac arrhythmias, myocardial infarction, and angina pectoris. It can also be used for preventive treatment of migraine [4].

Trimetazidine dihydrochloride (TMZ), (1-[(2,3,4-trimethoxyphenyl)methyl]-piperazine dihydrochloride) (Figure 1), is also an official drug in BP [1]. TMZ is used as an antianginal drug acting mainly on ischemic cells [5]. The mechanism of action is based on changing the energy substrate preference from fatty acid oxidation to glucose oxidation which adjusts the cellular energy processes, and in the main time it maintains appropriate energy metabolism during ischemia condition. It has no effect on the myocardial oxygen consumption or the coronary blood flow [6].

TMZ has no negative inotropic effects or vasodilatory characteristics and can be combined with other antianginal drugs as a complementary therapy mostly for patients with companion diseases such as left ventricular dysfunction and diabetes mellitus [7]. A comparison between ATN-TMZ combined therapy and atenolol plus placebo has been established and has shown an enhancement in the total exercise test duration and time to 1-mm ST segment depression. This combination was found to decrease the necessity of nitrate consumption, reduce the angina pain grade, and also decrease the overall number of angina attacks [7].

Coadministration of different medications for controlling chronic diseases especially in geriatric patients may cause polypharmacy problems such as adverse drug effects and toxicity. Changes in kidney and liver functions may affect drug pharmacokinetic and pharmacodynamics and may increase the mortality rates. Monitoring concomitant drugs in blood, plasma, and urine samples is mandatory in some cases [8]. Atenolol and trimetazidine can be coadministered due to their combined therapy benefits [7] and absence of interactions [9]. Doses may be adjusted to avoid any adverse effects with other drugs.

Atenolol experiences low or no hepatic metabolism and is excreted unchanged mainly in urine [4]. TMZ is weakly bound to plasma proteins. It is metabolized at a low extent into different metabolites and majority of the drug is excreted intact in urine [7]. Both analytes are considered good candidates for simple and rapid urine analysis, therapeutic monitoring, polypharmacy issues investigation, and toxicological studies. On the other hand, urine samples are noninvasive and easily accessible [10].

Different analytical methods have been applied for the determination of atenolol in biological fluids and pharmaceutical products such as HPLC [3, 11], LC-MS [5, 6], GC-MS [12], TLC [13], spectrophotometric and spectrofluorimetric methods [14–16], titrimetry [17], voltammetry [18], chemiluminescence [19], and capillary electrophoresis [20]. Several clean-up methods such as liquid-liquid extraction [3, 11, 12], solid phase extraction [21], and protein precipitation [22] have been used for extracting ATN from biological fluid samples.

Several techniques have been also reported for the determination of trimetazidine including HPLC [23, 24] in human plasma and dosage forms, respectively, LC-MS [5, 25], GC-MS [26, 27], HPTLC [28, 29], spectrophotometry [30, 31], potentiometry [32], voltammetry [33], and chemiluminescence [34]. TMZ has been isolated from biological fluids using liquid-liquid extraction [5], solid phase extraction [35], and protein precipitation methods [6].

To our knowledge, there are no published methods for determination of TMZ and ATN in human urine simultaneously. This paper describes simple HPLC-PDA methods for simultaneous determination of TMZ and ATN in human urine and also for determination of TMZ and ATN in their corresponding tablets.

## 2. Experimental

### 2.1. Materials and Reagents

All chemicals were of analytical grade and all solvents were of HPLC grade. Trimetazidine was kindly supplied from SIGMA Pharmaceutical Industries (Cairo, Egypt). Atenolol was kindly supplied from Egyptian Int. Pharmaceutical Industries Co. (10th of Ramadan city, Egypt). Metacardia® film-coated tablets labeled to contain 20 mg trimetazidine per tablet and Blokium® film-coated tablets labeled to contain 100 mg atenolol per tablet were obtained from local pharmacy. Acetonitrile, HPLC grade, orthophosphoric acid, and potassium orthophosphate monobasic were obtained from Fischer Scientific UK (Bishop Meadow Road, UK). Methanol and water were obtained from TEDIA Chemicals (USA). Sodium hydroxide, chloroform, and n-butanol were purchased from El-Nasr Pharmaceutical Chemicals Co. (Abu-Zaabal, Cairo, Egypt). Human blank urine was collected from healthy nonsmoker adult volunteers. Urine samples were stored at -20°C.

### 2.2. Instrumentation

The HPLC system consists of Agilent Technologies 1200 series chromatographic apparatus (Agilent Technologies, Palo Alto, CA, USA) equipped with an autosampler injector, 100 *μ*l injection loop, Agilent quaternary pumps, and solvent cabinets. Mobile phase was filtered through a 0.45 *μ*m membrane filter (Millipore, Ireland) and degassed with vacuum degasser. Separation was carried out on Caltrex BI (125 × 4.0 mm, 5 *μ*m) using UV lamp (Germany) and G1315D photodiode array detector (PDA).

### 2.3. Preparation of Stock Solutions

Stock solutions of either trimetazidine or atenolol were prepared individually at 1.0 mg/mL in methanol. Working solutions were prepared by further dilution of the stock solutions with water for spiking calibration standards and quality control samples. All stock solutions were stored in amber glass containers at 2-8°C.

### 2.4. Preparation of Calibration Standards and Quality Control Samples

#### 2.4.1. Neat Standard Solutions

Calibration standards were prepared in HPLC grade water at concentrations of 1.0, 3.0, 5.0, 10, 25, 50, 75, and 100 *μ*g/mL of both analytes. Low, med, and high quality control (QC) samples were prepared at concentrations of 3, 50, and 75 *μ*g/mL, respectively. 20 *μ*L of the prepared solutions were injected into the chromatographic system. Standard calibration curves were constructed by plotting peak area against the corresponding concentrations.

#### 2.4.2. Human Urine

Calibration standards were prepared in blank human urine at concentrations of 0.25, 0.5, 1.0, 10, 20, and 25 *μ*g/mL and of 0.5, 1.0, 10, 20, and 25 *μ*g/mL for TMZ and ATN, respectively. Low, med, and high quality control (QC) samples were prepared at concentrations of 0.5, 10, and 20 *μ*g /mL of TMZ and 1.0, 10, and 20 *μ*g /mL of ATN. Samples were stored at –20°C, extracted as described below, and calibration curves were constructed by plotting peak area against concentrations.

### 2.5. Preparation of Pharmaceutical Tablets Working Solutions

#### 2.5.1. Metacardia® Tablets

Five tablets were weighed, ground, and mixed well. An accurately weighed amount of powdered tablets equivalent to 50 mg of trimetazidine was extracted with 50 ml methanol and sonicated for 30 minutes. The solution was filtered and subsequent dilutions were made in water for HPLC-PDA analysis.

#### 2.5.2. Blokium® Tablets

Five tablets were weighed, ground, and mixed together. An accurately weighed amount of powdered tablets equivalent to 50 mg of atenolol was extracted with 50 ml methanol and sonicated for 30 minutes. The solution was filtered and the filtrate was subsequently diluted with water for HPLC analysis.

### 2.6. Human Urine Sample Preparation

Human urine samples were thawed at room temperature and mixed well. A 2.0 mL aliquot of each sample was transferred into screw cap culture tube, 0.5 mL of 1.0 N NaOH was added, and the tubes were vortex mixed for 30 seconds. Samples were extracted by adding 6.0 ml of (4:1) chloroform: n-butanol, v/v; samples were vortex mixed for 10 minutes. After centrifugation at 3000 rpm for 10 min, the organic layer was transferred into clean tube and evaporated to dryness under a nitrogen stream at approximately 40°C. The residue was reconstituted with 0.5 mL methanol, vortex mixed briefly. A 20 *μ*L of the final extract was injected into the HPLC system.

### 2.7. Chromatographic and Detection Conditions

#### 2.7.1. Neat Standard Solutions and Pharmaceutical Tablets

Chromatographic separation was carried out on a Caltrex BI (125 × 4.0 mm, 5 *μ*m) column. Mobile phase A consisted of 25mM potassium dihydrogen orthophosphate adjusted to pH 3.3 with orthophosphoric acid, mobile phase B was methanol, and mobile phase C was acetonitrile. The autosampler rinse solution was methanol. A gradient method at a flow rate of 1.0 mL/min was used, column temperature was maintained at RT, and total run time was 10. 0 minutes. The gradient elution was as follows: 0-1 min 10% B and 0% C, 1.1 min 20% B and 0% C, 4.0 min 15% B and 15 %C, 4.1 min 20 %B and 20%C, 8.0 min 80%B and 0% C, and 8.1-10.0 min 10% B and 0% C. Photodiode array detector (PDA) was operated at 210 and 225 nm for trimetazidine and atenolol, respectively.

#### 2.7.2. Urine Samples

Same conditions as described above were applied for the analysis of target analytes in urine samples except the following: the gradient was 0-2.0 min 10 %B and 0 %C, 2.1 min 20 %B and 0 %C, 5.0 min 15% B and 15 %C, 5.1 min 20 %B and 20%C, 5.1-8.0 min 80%B and 0%C, 8.0-11.0 min 90% B and 0% C, and 11.1-13.0 min 10% B and 0% C. Run time was 13.0 min.

## 3. Results and Discussions

### 3.1. Method Development

**Figure 2:**
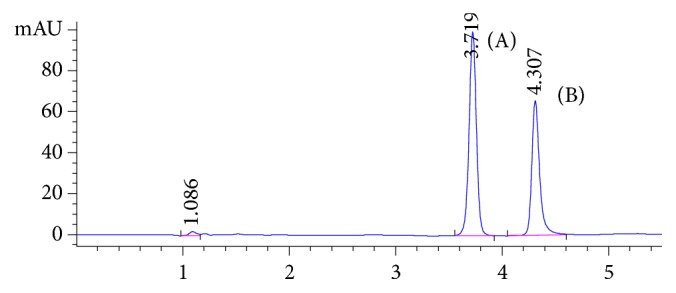
HPLC-PDA chromatogram of standard neat solution of (A) atenolol 10 *μ*g/mL and (B) trimetazidine 10 *μ*g/mL.

**Figure 3:**
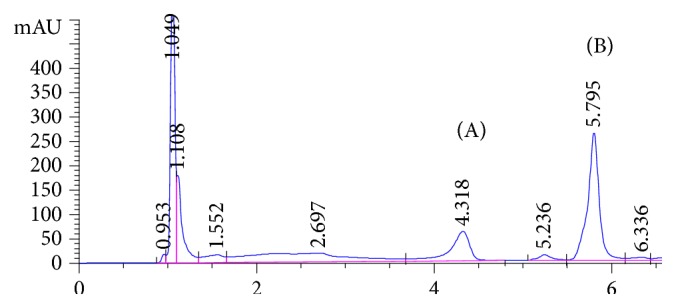
HPLC-PDA chromatogram of (A) atenolol 25 *μ*g/mL and (B) trimetazidine 25 *μ*g/mL in urine sample.

**Figure 4:**
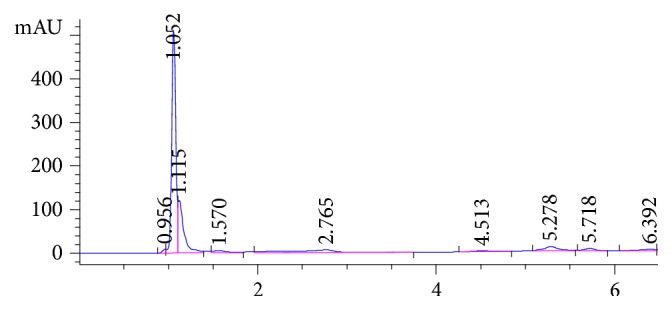
HPLC-PDA chromatogram of blank urine.

Target analytes exhibit different absorption characteristics, so 210 and 225 nm were selected for detection of TMZ and ATN, respectively. All chromatographic parameters were fully optimized to achieve the best chromatographic separation. Both methanol and acetonitrile were tested as organic modifiers in different proportions using both isocratic and gradient elution; methanol showed higher sensitivity but with asymmetric peak shape for both analytes. A gradient elution using both methanol and acetonitrile (as described above) showed excellent symmetric peak shape for both analytes. Addition of buffer modifiers (using different mobile phase buffers) and effect of mobile phase pH (pH 3-5) have been studied in terms of peak shape, elution time, and sensitivity. Lower pH mobile phase showed rapid elution; however, pH 5 showed late elution for both analytes. 25 mM phosphate buffer, pH 3.3, was found to be the most appropriate buffer modifier, providing well-resolved peaks in reasonable elution time. The flow rate range from 0.5 to 1.0 mL/min was tested. It was found that 1.0 mL/min was optimum for good chromatographic separation in a reasonable time without interference from either pharmaceutical preparation placebo components or biological fluid endogenous peaks. Different gradient programs have been applied; peak symmetry, resolution, selectivity, and number of theoretical plates were calculated in each case and summarized in Table 1.  Figure 2 shows a typical chromatogram for a laboratory prepared mixture of the studied drugs under the optimized chromatographic conditions. The retention times for atenolol and trimetazidine were 3.72 and 4.3 minutes, respectively. Due to the nature of biological fluid extracts and presence of extracted endogenous components, the gradient has been slightly modified for urine sample analysis. Longer desalting step at high percentage of aqueous mobile phase was applied to ensure complete removal of polar components before elution of target analytes. Also, the column wash step using high percentage organic mobile phase was extended in order to elute any endogenous matrix components in the final extracts and avoid any coelution of late eluting peaks with target analytes in subsequent injections.  Figure 3 represents the chromatogram of the analytes in urine samples showing that ATN and TMZ were eluted at 4.32 and 5.8 minutes, respectively, and Figure 4 shows the blank urine chromatogram in which there was no interference at the elution times of our analytes.

Liquid-liquid extraction using organic solvents has been shown to provide clean extracts of biological fluid samples; it is also considered a cheaper sample treatment approach. Different organic solvents (dichloromethane, chloroform, n-hexane: ethyl acetate (1:1, v/v), and chloroform: n-butanol (4:1, v/v)) have been evaluated for extraction recoveries. It was found that 4:1 chloroform: n-butanol, v/v, not only provided the highest extraction recoveries for both analytes but also eliminated the interference of endogenous matrix components. pH of the sample is known to strongly affect the ionization of different compounds and subsequently affect the extracted portion of the analyte into the organic layer; acidic and basic conditions using 1.0 N HCl and 1.0 N NaOH, respectively, and matrix samples without any pH optimization (using water) have been compared in terms of extraction recoveries and cleanness of the final extracts. Both target drugs are basic compounds (of PKa 9.6 and 9.14 for ATN [36] and TMZ [37], respectively); their extraction recoveries were significantly improved by raising the pH of the sample with sodium hydroxide solutions. Different volumes of 1.0 N NaOH were tested; a 500 *μ*l aliquot of 1.0 N NaOH provided the best extraction recoveries. Extraction recoveries were found to be 45% and 100% for ATN and TMZ, respectively, and were consistent against different levels for both analytes.

System suitability parameters such as resolution (Rs), tailing factor (T), capacity factor (K'), and selectivity factor (*α*) were calculated according to USP [2] as shown in Table 2. The resolution was always more than 1.5, the selectivity was more than one, and the symmetry factor had an accepted value.

*Linearity*. Peak area responses were plotted against the corresponding concentrations. Linearity was obtained over a concentration range of 1.0-100 *μ*g/mL in pure form for both analytes and over concentration ranges of (0.25-25) *μ*g/mL and (0.5-25) *μ*g/mL in urine samples for trimetazidine and atenolol, respectively. The correlation coefficient was ≥ 0.999 as shown in Table 1.

*Limit of Detection and Limit of Quantitation*. The limit of detection (LOD) is the lowest concentration at which the analyte can be detected. The limit of quantitation (LOQ) is the lowest concentration that can be quantitatively measured with acceptable precision and accuracy. The LOD was established as the lowest concentration that provides a signal-to-noise (S/N) ratio of 3:1 and the LOQ was established as the lowest concentration that provides S/N ratio of 10:1 as given in (Table 1).

*Statistical Analysis for Pure Form Method*. The proposed method was compared to the official and reported methods [1, 38] statistically using Student's* t*-test and variance ratio F-test at 95% confidence level. The calculated* t* and F values did not exceed the theoretical values. No significant differences between the proposed method and the official or reported ones have been observed as given in (Table 3). 

*Precision and Accuracy*. Inter- and intraday precision (measured as percent relative standard deviation, %RSD) and inter- and intraday accuracy (measured as percent difference from the nominal concentration, %DFN) were measured at low, mid, and high QC levels. Excellent precision and accuracy were obtained for both analytes in pure forms and urine samples (Tables 4 and 5). 

*Selectivity*. Selectivity is the ability of the analytical method to determine the target compound in the presence of other components such as excipients, degradation products, coadministered drugs, and/or endogenous matrix components without interference. The proposed method showed excellent chromatographic separation and quantitation of the cited drugs in the presence of common tablet excipients and urine extracted matrix. No significant peaks were detected at the expected retention times of the two cited drugs, and there is no coelution with any of the other components that may present in the final extracts. 

*Robustness*. Small variations in the experimental parameters were applied and the effect of these variations on the analytical performance was evaluated to ensure the robustness of the proposed method. The studied parameters were as follows: phosphate buffer pH (3.3 ± 0.2), flow rate (1.0 ± 0.01) mL/min, and wavelength 210 nm ±1.0 and 225 nm ±1.0 for TMZ and ATN, respectively. Only one parameter was changed in a time and the others were kept unchanged. The effects of the proposed changes on retention time, peak area, tailing factor, and number of theoretical plates were evaluated. It was found that all the studied minor changes have no significant effects on the method performance. 

*Applications*

*Pharmaceutical Tablets*. The optimized HPLC method was successfully applied for determination of the studied drugs in their corresponding tablets. Good percentage recoveries without any excipients interference were obtained (Table 6); the proposed method can be applied for quality control studies.

## 4. Conclusion

RP-HPLC method with PDA detection was developed for the determination of atenolol and trimetazidine in pure form, pharmaceutical tablets, and human urine. The proposed method was found to be simple, sensitive, and accurate. The optimized chromatographic conditions allowed separation of the studied drugs in reasonable time without any interference from excipients and/or extracted matrix components. This method can be used for therapeutic drug monitoring of patients treated with ATN and TMZ concomitantly and also can be applied for quality control analysis of the studied drugs in their pharmaceutical tablets.

## Figures and Tables

**Table 1:** Analytical performance data for HPLC determination of atenolol and trimetazidine in pure form and urine samples.

Parameter	Atenolol		Trimetazidine	
	Pure form	Urine	Pure form	Urine
Linearity range (*μ*g/ml)	1.0–100	0.5-25	1.0– 100	0.25-25
Correlation coefficient (r^2^)	0.9998	0.9999	0.9999	0.9995
Regression equation	Y=39.15X-5.7394	Y=0.037X-8.0947	Y=118.06X-58.05	Y=0.3464X+31.83
LOD (*μ*g/ml)	0.30	0.11	0.18	0.05
LOQ (*μ*g/ml)	0.99	0.38	0.60	0.16

**Table 2:** System suitability parameters for the determination of the atenolol and trimetazidine in pure form/human urine.

Parameters	Atenolol	Trimetazidine	Reference value [39, 40]
Retention time (t_R_)	3.72/4.32	4.31/5.79	-----
Number of theoretical plates (N)	13211/2896	13935/13668	>2000
			increase with efficiency of separation
Tailing factor (T)	0.97/1.85	0.71/1.11	≤2
Capacity factor “Mass distribution ratio” (K′)	2.44/2.32	2.98/3.46	1-10 acceptable
Height equivalent to one theoretical plate (HETP)	0.01/0.04	0.01/0.01	The smaller the value, the higher the column efficacy
Resolution (R_s_)	1.62/4.89		>1.5
			Good separation between peaks of interest.
Selectivity factor (*α*)	1.11/1.49		> 1

**Table 3:** Statistical analysis of the proposed method in standard solution and the official/reported methods [1, 38].

	Atenolol		Trimetazidine	
	Proposed Method	Reported method [38]^*∗∗*^	Proposed Method	Official method [1]
Mean	100.34	99.66	100.35	98.39
SD	1.17	0.87	1.06	0.77
RSD%	1.16	0.88	1.06	1.12
Variance	1.36	0.76	1.13	0.59
N	8	5	8	3
F – test	1.8 (4.12)^*∗*^	-	1.09 (4.74)^*∗*^	-
Student's *t-test*	1.11 (2.201)^*∗*^	-	1.93 (2.262)^*∗*^	-

^**∗**^Figures between parenthesis represent the corresponding tabulated values of *t* and F at P= 0.05.

^*∗∗*^Spectrophotometric method based on measurement of atenolol absorbance in 0.1 N HCl at 224.6 nm.

**Table 4:** Intra- and interday precision and accuracy calculated from quality control (QC) samples in pure form.

**QC conc. (*μ*g/mL)**	**Intra-day**				**Inter-day**			

	**Mean (*μ*g/mL)**	**SD**	%**RSD**	%**DFN**	**Mean (*μ*g/mL)**	**SD**	%**RSD**	%**DFN**

**ATN**								
3	2.97	0.02	0.77	-1.12	3.01	0.05	1.7	0.25
50	50 .95	0.45	0.88	1.89	50.17	0.41	0.82	0.34
75	75.004	0.12	0.17	0.005	76.36	0.29	0.39	1.82
**TMZ**								
3	2.89	0.09	3.45	-3.67	2.88	0.09	3.02	-3.99
50	50.09	0.23	0.45	0.18	50.07	0.26	0.52	0.15
75	77.14	1.31	1.7	2.85	76.34	2.05	2.68	1.78

**Table 5:** Intra- and interday precision and accuracy calculated from quality control (QC) samples in human urine.

	**Intra-day **				**Inter-day **			

	**Mean (*μ*g/mL)**	**SD**	%**RSD**	%**DFN**	**Mean (*μ*g/mL)**	**SD**	%**RSD**	%**DFN**

**ATN**								
**1.0**	1.00	0.02	1.76	0.08	0.99	0.03	2.85	-0.01
**10**	9.92	0.07	0.73	-0.81	9.85	0.11	1.10	-1.50
**20**	20.02	0.25	1.26	0.11	20.08	0.17	0.82	0.41
**TMZ**								
**0.5**	0.50	0.004	0.75	0.98	0.50	0.004	0.69	0.90
**10**	10.19	0.01	0.98	1.9	10.14	0.12	1.18	1.37
**20**	19.36	0.08	0.42	-3.19	19.41	0.08	0.42	-2.94

**Table 6:** Application of the proposed method in standard solution on pharmaceutical tablets.

**Pharmaceutical Preparations**	**Blokium® tablets**			**Metacardia® tablets**		
	**Taken (*μ*g/mL)**	**Found** ^**∗**^ ** (*μ*g/mL)**	**Recovery **%	**Taken (*μ*g/mL)**	**Found** ^**∗**^ ** (*μ*g/mL)**	**Recovery **%
	**5.00**	4.87	97.44	5.00	4.73	94.54
	**10.00**	9.60	95.97	10.00	9.74	97.40
	**30.00**	28.63	95.42	30.00	28.50	95.05
	**50.00**	47.50	95.01	50.00	49.15	98.30
	**75.00**	71.67	95.56	75.00	71.24	94.99
	**100.00**	95.27	95.27	100.00	95.36	95.36
**Mean**			95.78			95.94
**SD**			0.88			1.53
**SE**			0.36			0.63
**RSD**			0.91			1.60
**Variance**			0.77			2.34

^**∗**^Average of three determinations.

## Data Availability

The data used to support the findings of this study are available from the corresponding author upon request.
